# Utilization of preimplantation genetic testing in the USA

**DOI:** 10.1007/s10815-021-02078-4

**Published:** 2021-04-26

**Authors:** Kaitlyn Roche, Catherine Racowsky, Joyce Harper

**Affiliations:** 1 University College London EGA Institute for Women’s Health, London, UK; 2 Hospital Foch, Suresnes, France and Brigham and Women’s Hospital, MA Boston, USA

**Keywords:** PGT, PGT-A, SART, CDC, Preimplantation diagnosis

## Abstract

**Purpose:**

To evaluate the use of preimplantation genetic testing (PGT) and live birth rates (LBR) in the USA from 2014 to 2017 and to understand how PGT is being used at a clinic and state level.

**Methods:**

This study accessed SART data for 2014 to 2017 to determine LBR and the CDC for years 2016 and 2017 to identify PGT usage. Primary cycles included only the first embryo transfer within 1 year of an oocyte retrieval; subsequent cycles included transfers occurring after the first transfer or beyond 1 year of oocyte retrieval.

**Results:**

In the SART data, the number of primary PGT cycles showed a significant monotonic annual increase from 18,805 in 2014 to 54,442 in 2017 (*P* = 0.042) and subsequent PGT cycles in these years increased from 2946 to 14,361 (*P* = 0.01). There was a significant difference in primary PGT cycle use by age, where younger women had a greater percentage of PGT treatment cycles than older women. In both PGT and non-PGT cycles, the LBR per oocyte retrieval decreased significantly from 2014 to 2017 (*P*<0001) and younger women had a significantly higher LBR per oocyte retrieval compared to older women (*P* < 0.001). The CDC data revealed that in 2016, just 53 (11.4%) clinics used PGT for more than 50% of their cycles, which increased to 99 (21.4%) clinics in 2017 (*P*< 0.001).

**Conclusions:**

A growing number of US clinics are offering PGT to their patients. These findings support re-evaluation of the application for PGT.

**Supplementary Information:**

The online version contains supplementary material available at 10.1007/s10815-021-02078-4.

## Introduction

After the first two reports of world data on PGT in the 1990s [1, 2], the ESHRE PGT Consortium was established in 1997 to collect global PGT data [3]. The outcome of individual embryos following an ART treatment can be tracked from start to finish in the newer databases [4] and data are reported by indication, including the PGT subgroups (PGT for monogenic diseases (PGT-M), PGT for structural abnormalities (PGT-SR), and PGT for aneuploidy (PGT-A) [5]. This gives a very rich picture of the use of PGT. However, a considerable limitation of these data is that it only represents a small proportion of the global PGT performed. The latest ESHRE PGT Consortium paper contains data from only 2 US clinics [4].

In the USA, PGT data are collected and reported by the Society for Assisted Reproductive Technology (SART) and also reported by the Centers for Disease Control and Prevention (CDC). In both sets of data, PGT cannot be separated into PGT-A, M, or SR. In the UK, PGT data are collected by the Human Fertilisation and Embryology Association (HFEA) and are separated into PGT-A and PGT-M/SR. A recent analysis of the UK HFEA data and US SART data for 2014–2016 showed that in the UK, PGT was used in less than 2% of cycles, while in the USA, it was used in 21% of cycles [6]. Over these 3 years, 94,935 cycles using PGT were performed in the USA which is more than all the ESHRE PGD Consortium cycles collected since 1997.

The present study investigated the utilization and live birth rates of PGT in the USA among different age groups, as well as the use of PGT at both clinic and state levels.

## Materials and methods

### Data collection

Data were drawn from the US national reports of SART for 2014 to 2017 and the CDC for 2016 and 2017. Both data sources were accessed online to investigate the use of PGT for autologous cycles and so institutional review board (IRB) approval was not required. Because the CDC combined reporting for autologous and oocyte donor cycles prior to 2016, the CDC analysis was restricted to 2016 and 2017 for the purpose of this study.

Due to the differences in inclusion criteria for cycles between the two databases, these two data sources were not directly compared. Individual analyses were performed using each database, where SART data were evaluated for trends in overall ART and PGT use for autologous cycles, while the CDC data were evaluated for trends in the use of PGT across individual clinics and states.

### SART

PGT usage is available from SART in the SART CORS registry from 2003 to 2017 (www.sart.org). However, for the purpose of this project, only cycles from 2014 to 2017 were analysed. SART CORS collects data from more than 95% of clinics offering fertility services in the US (www.sart.org).

The interactivity of the SART database allowed for filters to be applied to accurately differentiate between those cycles that used PGT from those that did not. Live birth rates were determined for PGT versus non-PGT cycles, where live birth rate was defined as the percentage of oocyte retrievals or embryo transfer procedures that led to the birth of at least one living child [7].

Both fresh and frozen autologous cycles were included and defined as either primary or subsequent. A primary cycle was counted when the first embryo transfer procedure occurred within a year of the oocyte retrieval. A subsequent cycle was defined as an embryo transfer (from thawed oocytes or embryos) that occurred 1 year or more after the oocyte retrieval or if this was not the first embryo to be transferred [7].

### CDC

The CDC Fertility Clinic Success Rates Reports included data from all reporting SART clinics, as well as clinics that were not SART members (https://www.cdc.gov/art/reports/2016/fertility-clinic.html). The CDC report included about 98% of ART cycles in the USA [8].

Although CDC data were available from 2004, we only included data from 2016 and 2017 because prior to 2016, PGT usage was only reported for fresh, autologous oocyte cycles. In 2016, the reports were restructured to include both autologous and donor oocyte cycles together, except for cycles started with the intention of banking oocytes and cycles that involved experimental procedures [9].

Due to the PDF format of the CDC report, the information could not be filtered. Therefore, live birth rates, age profiles, and information on the number of embryos in a cycle could not be assessed. Nevertheless, the reports enabled the evaluation of the percentage of cycles within each clinic using PGT. Overall PGT use by state was also determined.

### Statistical analysis

For trends over time, the non-parametric Kendall’s tau correlation coefficient was determined on the SART data to determine if these trends were significant. To identify differences between percentages in age groups and 4 years of interest, a repeated-measures analysis of variance (ANOVA) was, if significant, followed by the post hoc Bonferroni tests for pairwise comparisons. The latter adjusted the *P* values to avoid spuriously significant results arising from multiple comparisons. The assumptions of the ANOVA were verified by a study of the residuals. Chi-squared tests were used to assess associations between years (2016 and 2017) and percentage use of PGT using the CDC data. Results were classified as significant when a *P* value less than 0.05 was obtained. SPSS (IBM Corp. Released 2020. IBM SPSS Statistics for Windows, Version 27.0. Armonk, NY: IBM Corp) was used for all statistical analyses.

## Results

### PGT use by cycle—SART

The total number of ART cycles per year demonstrates a significant monotonic annual increase from 140,392 in 2014 to 171,381 in 2017 (Table 1, Kendall’s tau = 1.0, *P=*0.042). The number of PGT cycle and the number of PGT cycles expressed as a percentage of the number of ART cycles both showed a significant monotonic annual increase from 18,805/140,392 (13%) in 2014 to 54,442/171,381 (32%) in 2017 (Table 1, Kendall’s tau = 1.0, *P* = 0.042)

**Table 1:** SART data analysis of the number of ART, non-PGT, and PGT cycles (primary and subsequent) from 2014 to 2017

Year	Total ART cycles	Total number non-PGT cycles (% of total ART cycles)	Total number of PGT cycles (% of total ART cycles)	Total subsequent cycles	Number subsequent non-PGT cycles (% of total)	Number of subsequent PGT cycles (% of total
2014	140,392	121,587 (87%)	18,805 (13%)	32,381	29,435 (91%)	2946 (9%)
2015	154,490	121,921 (79%)	32,569 (21%)	37,709	31,583 (84%)	6126 (16%)
2016	166,540	120,160 (72%)	46,380 (28%)	42,272	32,535 (77%)	9737 (23%)
2017	171,381	116,939 (68%)	54,442 (32%)	47,166	32,805 (70%)	14,361 (30%)
Total	632,803	480,607 (76%)	152,196 (24%)	159,528	126,358 (72%)	33,170 (20%)

In 2014, 2946 subsequent PGT cycles were performed. This number increased monotonically to 14,361 cycles in 2017 (Table 1, Kendall’s tau =1.0, *P*= 0.042). This was equivalent to a 4.87-fold increase (14,361/2,946) in 4 years

#### Age profiles

There was no evidence of an interaction between year and age group in the repeated measures ANOVA of the percentage, in a given year, of PGT cycles by age group. The percentage of PGT treatment cycles performed within each age group did not significantly change over time (ANOVA, *P*=0.99) (Supplementary Figure 1). However, the percentage of PGT cycles performed varied significantly by age group (ANOVA, *P*<0.001). The Bonferroni post hoc pairwise comparisons indicated that the percentage was significantly different in all age groups (*P* < 0.001) apart from the comparison of age 35–37 years with 38–40 years (*P* > 0.999), with younger women having a greater percentage of PGT treatment cycles than older women.

#### Live birth rates

**Fig. 1:**
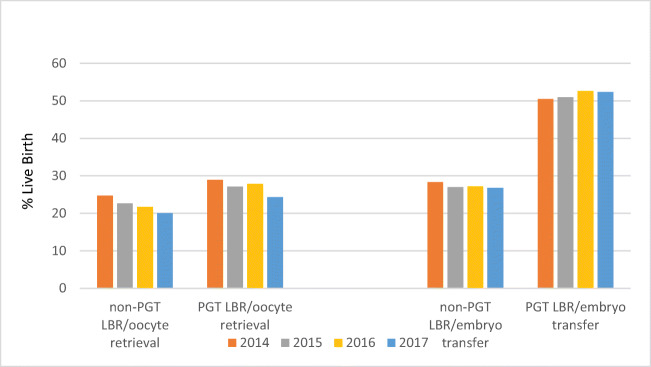
The LBR for non-PGT and PGT per oocyte retrieval and transfer procedure from 2014

When combining all age groups together, the LBR per oocyte retrieval decreased significantly between 2014 and 2017 for both non-PGT (25 to 20%) and PGT cycles (29 to 24%) (*P* = 0.002), both reaching their lowest points in 2017 (Fig. 1). The LBR per embryo transfer for PGT was significantly higher than that for non-PGT for all 4 years (*P*<0.01).

### Live birth rates by age

#### Primary cycles

**Fig. 2:**
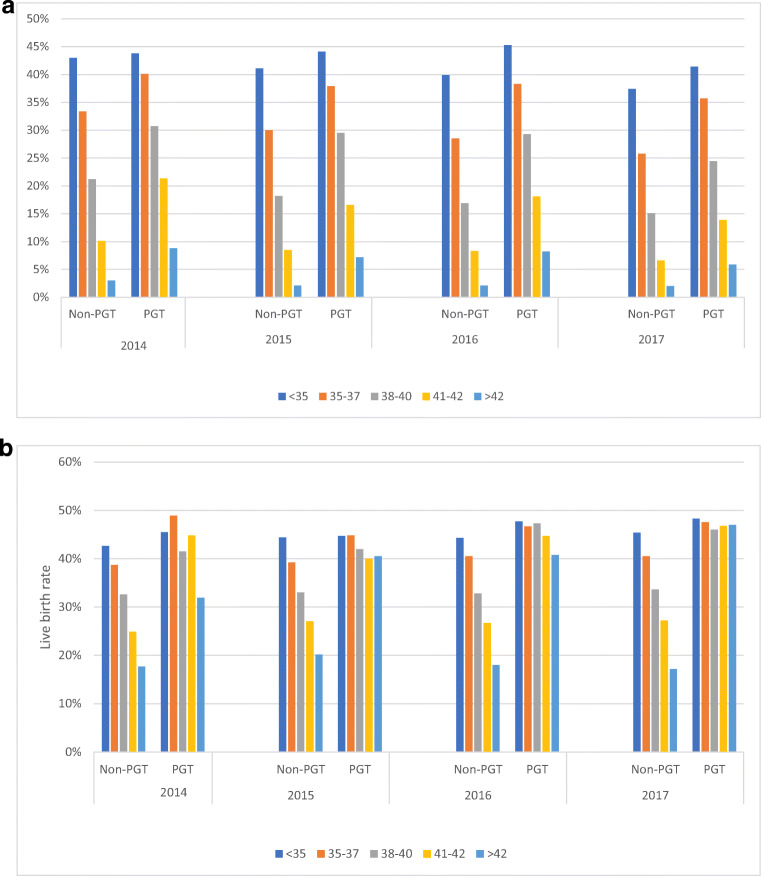
**a** The LBR per oocyte retrieval for primary non-PGT and PGT cycles stratified by patient age from 2014 to 2017. **b** LBR for subsequent non-PGT and PGT cycles from 2014 to 2017

Figure 2a shows the LBR per oocyte retrieval from 2014 to 2017 by age group for primary cycles for both non-PGT and PGT cycles. There was no evidence of an interaction between year and age group in the repeated measures ANOVA of the PGT LBR per oocyte retrieval. Post hoc comparisons following the significant ANOVA test for the comparisons of the LBR in the 4 years (*P* < 0.001) indicated that the PGT LBR per oocyte retrieval was significantly greater in 2014 than in 2017 (*P* < 0.001) but there was no evidence of a difference between the PGT LBR in any other years (*P* > 0.05). The Bonferroni tests following the significant ANOVA test for the comparison of the PGT LBR in the five age groups (*P* < 0.001) indicated that the LBR differed significantly between all age groups (*P* < 0.001), with a greater LBR in younger compared to older age groups. For the non-PGT cycles, there was no evidence of an interaction between year and age group in the repeated measures ANOVA of the LBR per oocyte retrieved. Post hoc comparisons following the significant ANOVA test for the comparison of the non-PGT LBR in the 4 years (*P* < 0.001) indicated that the non-PGT LBR per oocyte retrieval was significantly greater in 2014 than in any other year and that it was significantly greater in 2015 than in 2017 but there was no evidence of a difference between the LBR in any other years (*P* > 0.05). Post hoc comparisons following the significant ANOVA test for the comparison of the non-PGT LBR in the five age groups (*P* < 0.001) indicated that the non-PGT LBR per oocyte retrieval was significantly different in all age groups (*P*< 0.001) with a monotonic downward trend in the LBR from the youngest to the oldest age groups in every year.

#### Subsequent cycles

The LBR for subsequent PGT cycles was higher for all age groups over all four years compared to the LBR of subsequent non-PGT cycles (Fig. 2b).

The subsequent PGT cycles resulted in a consistent increase in LBR for all age groups across the four study years. While there was observable variability between age groups in 2014, the LBR from subsequent PGT cycles is more comparable between age groups after 2014, until there was little noticeable difference in 2017 (Fig. 2b). This convergence was, in part, due to the increase in LBR for subsequent PGT cycles for patients over the age of 42.

#### Clinic use of PGT—CDC

**Fig. 3:**
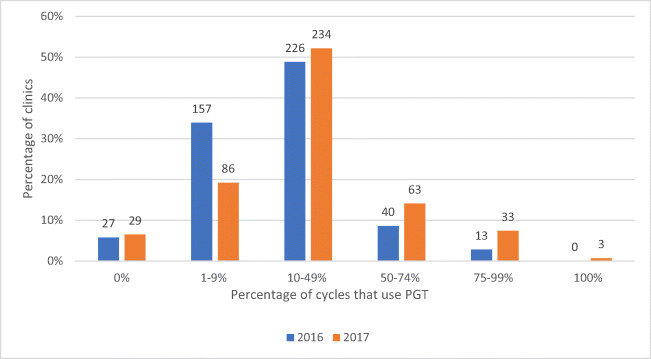
Comparison of PGT usage by US clinics in 2016 compared with 2017. Numbers above the bars are the number of clinics

Figure 3, with data from the CDC National Fertility Clinic Success Rate Report, illustrates the number and percent of clinics according to categories of PGT use in 2016 and 2017. There was a significant association between these factors (chi-squared test *P* < 0.001). Furthermore, in 2016, just 53 (11.4%) clinics used PGT for more than 50% of their cycles which increased to 99 (22.1%) clinics in 2017 (chi-squared test *P* < 0.001). While there were no clinics that used PGT for 100% of their cycles in 2016, the number increased 3 (0.67%) in 2017.

Of states with the highest percentage of PGT use, New Mexico, Delaware, Mississippi, California, and Oregon remained in the top 5 in both 2016 and 2017 (Supplemental Table 1A) with each state increasing its percentage of PGT use from 2016 to 2017. Notably, the highest percentage of PGT use for a state (New Mexico) in 2016 was 70.5% while that number increased by 9.9 to 80.4% in 2017.

Similarly, of the states with the lowest percentage of PGT use, Arkansas, West Virginia Puerto Rico, Vermont, Montana, and North Dakota remained in the bottom states for both 2016 and 2017 (Supplemental Table 1B). The state with the lowest percentage of PGT use in 2016 (1.0%) was Arkansas whereas, in 2017, it was Puerto Rico (3.3%). There was very little change in the percentage of PGT use in any of these states between 2016 and 2017.

The states that showed the greatest increase in the percentage use of PGT were Idaho, Hawaii, South Carolina, Kentucky, and Missouri (Supplemental Table 1C) with a mean increase of 17.5% (95% CI 15.3 to 19.8%).

## Discussion

It is important to monitor the use of ART and PGT cycles globally. Since the first two world data reports [1, 2], the ESHRE PGD Consortium has been reporting annual data but the latest report for data I–XV (1997–2013) only contains a total of 56,093 PGT cycles, which includes 32,832 PGT-A, 14,340 PGT-M, and 8921 PGT-SR [4] and data from just two US clinics. In this report, we have determined that in just 4 years 152,196 primary and 33,170 subsequent cycles of PGT have been carried out in the USA. With the caveat that there are limitations to analysing large data sets, as discussed below, the data presented here reports some key trends for the use of PGT in the USA.

A previous report covering cycles from 2014 to 2016 showed a much higher percentage of PGT in the USA (24%) compared to that in the UK (< 1%) and the possible reasons for this were explored [6]. Here, we report a continued increase in the use of PGT in the USA. From 2014 through to 2017, the number of PGT cycles significantly increased, reaching an all-time high of 32% of ART cycles in 2017.

It is unsurprising that there has been an increase in subsequent cycles because many women undergo numerous stimulation cycles in order to bank embryos that can be used for PGT. Having a larger number of embryos from multiple oocyte retrievals may increase the chance of pregnancy per transfer, but not per oocyte retrieval. The high proportion of frozen PGT cycles is also likely to reflect the trends towards next-generation sequencing which is more efficient and cost-effective if embryos are frozen as it allows more time for the diagnosis and samples can be batched reducing the cost [10, 11].

In this study, PGT is 5 times more likely to be undertaken in women under the age of 35 years which is in agreement with a previous study [6]. There could be several reasons for this, including that younger women produce more embryos and so may fit the criteria for PGT and that PGT is often offered after a failed cycle. Older women may be less likely to embark on further IVF after a failed cycle. Several RCTs have been performed on women of advanced maternal age [12] as they are at most risk of having chromosome abnormalities in their embryos, but studies on younger women have been conducted [13, 14]. RCTs on PGT-A to date have failed to identify the patients best suited for PGT-A treatment. Further studies are urgently needed to determine which age groups might benefit.

### Live birth rates

A limitation of the analysis of the data we present is that it does not adequately control for confounders. A recent study found that ART adjuncts are likely contributing to a decline in ART LBRs [15]. This is also in line with the statement by the American Society for Reproductive Medicine and the Society for Assisted Reproductive Technology, which advises that PGT-A does not improve ART outcomes [16]. Further in-depth analyses are required to understand these data further.

It has been suggested that PGT-A will reduce the time to pregnancy, decrease miscarriage rates, and decrease the cost of ART as less ART cycles will be needed. The ESTEEM study showed that PGT-A patients had fewer transfers, fewer miscarriages, and fewer cryopreserved embryos but the same LBR as the control group [17]. The STAR trial showed no overall improvement in ongoing pregnancy rates at 20 weeks. Subgroup analysis of the women aged 35–40 years did show an increase in ongoing pregnancy rate if two or more embryos were biopsied, but these data were not significant when analysed by intention-to-treat and there was no effect on miscarriage rates [18]. Studies have shown that PGT cycles result in a lower number of frozen embryos, which will result in a lower cumulative live birth rate [17–19]. For both the ESTEEM and STAR trials, the additional births from frozen embryos have not yet been considered and may result in a lower live birth rate in the PGT-A group as less embryos are frozen after PGT. Only the ESTEEM study on polar body biopsy has shown an effect on miscarriages [17]; the STAR trial did not [18].

LBRs are influenced by the denominator used. Clearly, if calculated by the number of cycles started, this will be lower than when calculated by the number of oocyte retrievals which, in turn, will be even lower than when the denominator is the number of embryo transfers. For both PGT and non-PGT, the LBR per embryo transfer is higher than the LBR for oocyte retrieval (Fig. 2). This is because the LBR per transfer only comes into effect if there is a transfer procedure whereas LBR per retrieval and cycle started has to take into account, respectively, cancelled cycles and those with no embryos to transfer.

### Subsequent cycles

The number of subsequent PGT cycles increased significantly from 2014 to 2017, with the greatest increase occurring from 2016 to 2017. This increase is unsurprising because many women undergo numerous stimulation cycles to bank embryos that can be used for PGT, which has been shown to increase the chances of having an embryo transfer.

Subsequent cycles are an embryo transfer, from thawed oocytes or embryos, that occurred 1 year or more after the oocyte retrieval or if this was not the first embryo to be transferred. The increase in the number of subsequent PGT cycles could also be explained by the large number of clinics that perform ‘freeze all’ or ‘embryo banking’ cycles, where all oocytes or embryos that result from the initial oocyte retrieval are immediately frozen for later use [20, 21]. Some studies have shown that transferring a thawed embryo results in a significantly higher live birth rate [11, 22, 23] but a recent multicentre randomised controlled trial has shown no benefit [24]. Some clinics may offer to freeze all cycles in association with non-PGT and PGT.

Perhaps the most significant result shown in this study is that the LBR by age for subsequent cycles by PGT showed no evidence of an association between age and LBR, especially for the data for 2017. In the Theobald study, the same result was seen for the HFEA data but not the SART data when considering years 2014–2016. This finding agrees with the hypothesis that PGT-A is used to balance out LBRs across age groups [25].

### PGT use by state—CDC

The CDC data from 2016 and 2017 include 48 reporting states, including US territory Puerto Rico and the District of Columbia (Washington, D.C.). In both 2016 and 2017, there were no clinics in New Hampshire that reported data. Alaska does not have any fertility clinics. Analysis of the data showed that more clinics were using more PGT, giving a significant overall increase in PGT use from 2016 to 2017.

Cost plays a large role in the accessibility of PGT, which makes it important to understand the extent to which PGT is covered by insurance. Despite infertility being registered as a disease by the World Health Organization in 2009, most insurance plans fail to recognize it in their policies [26].

When assessing PGT use by state, New Mexico, Delaware, Mississippi, California, and Oregon were found to have the highest average PGT use in both 2016 and 2017 (Supplemental Table 1). Interestingly, both Delaware and California, amongst other states, have passed state laws that require insurance companies to cover infertility treatment. The California state law declares that insurers must cover treatment for infertility, indiscriminate of age, gender, marital status, etc. However, the law excludes coverage for ART treatment, and therefore PGT [27]. This means that the high number of PGT treatments that California performs is self-funded. Delaware’s state law, which was introduced in 2018, is more extensive and includes ART and gamete cryopreservation. This law includes a variety of ART add-ons, including PGT, which may explain why it has the second-highest PGT use of all reporting states in 2016 and 2017 [27].

In contrast, New Mexico and Oregon do not have state laws that require insurance companies to cover the cost of infertility treatments, which means that, like California, the high percentage of PGT cycles is self-funded.

Four of the states with the lowest average percentage of PGT use were also recurring between 2016 and 2017. The states that were consistently in the bottom include Puerto Rico, West Virginia, Arkansas, and Vermont (Supplementary Table 1C). West Virginia and Arkansas are both governed by laws that require coverage for infertility services. The law in Arkansas includes a lifetime maximum of $15,000, including for the use of ART. The law allows individual insurers to decide which treatments they cover. Therefore, the coverage of PGT varies. It is likely that PGT is not frequently covered by insurance, due to the low percentage of cycles that use PGT in the state of Arkansas. Similarly, West Virginia state law requires infertility services to be covered, but does not define the extent to which they must be covered. Therefore, it is likely that PGT is not covered by insurance, leading to the low percentage of cycles that use PGT in West Virginia [27]. Neither Puerto Rico nor Vermont has laws that require insurers to cover the costs of infertility services.

Of the states that showed dramatic increases in the percentage of cycles that used PGT, the only state that legally requires coverage is Hawaii. However, the law was last amended in 2003, so it would not have caused a difference between 2016 and 2017. Of the states with the smallest increase, or decrease, in the percentage of cycles that used PGT, Rhode Island, Maryland, and Louisiana all have laws that require the coverage of infertility services. However, all laws were put into place before 2016 and are unlikely to have affected change between 2016 and 2017 [27].

Partly due to the liberal approach to PGT, the USA has become a popular destination for reproductive tourism, in which people travel to the USA to receive treatments that are not available in their home countries. In 2013, PGT was reported in 19.1% of non-US resident cycles versus 5.3% of US resident cycles [28]. This likely affects the calculations of PGT use in clinics. This is also likely to contribute to the increased percentage of PGT use in the USA.

## Limitations

There are several limitations to this study. One of the main limitations is that PGT is a rapidly evolving area. From the data we analysed, it was not possible to determine the stage of biopsy or the method of diagnosis, something which the ESHRE PGT Consortium data does achieve. Also, with the SART data used in this study, it was not possible to differentiate between PGT-A, PGT-M, and PGT-SR. We have assumed that the vast majority of PGT cycles in the USA will be for PGT-A, as has been the case since 1997 in the ESHRE PGD Consortium data.

Another limitation in the SART data is the lack of clarity in the subsequent cycle data. It is unclear whether the patient’s age is reflective of the time of her oocyte retrieval or the time of cycle start. For this contribution, it was assumed that the age reflected the age at the time of oocyte retrieval. If not the case, this would likely have had a minor impact on the LBR results stratified by age. Subsequent data is also difficult to interpret as we cannot link cycles so we do not know what happened in the fresh cycle; a transfer, a pregnancy?

Although the CDC and SART work together to provide accurate reports of ART data, the difference between the two organizations is that while clinics are required by law to report their data to the CDC, there is no law in place that requires fertility clinics to become members of SART. While 83% of US clinics are members of SART, about 17% of clinics remain independent and therefore are not required to follow SART guidelines [29]. Furthermore, not all clinics submit their data to the CDC. The few clinics that do not comply are listed as ‘did not submit’ in the final reports, but they are not penalized in any way. This could be seen as a limitation with the government collection system.

## Conclusion

PGT use is continuing to increase in the USA. However, the LBR did not increase consistently with this observed growth.

PGT technology has drastically changed over the last few decades and there is no doubt it will continue to advance along with breakthroughs in science and the technique. Novel advancements have made PGT use easier than ever before, but it is undeniable that much more research, specifically RCTs, needs to be done to evaluate the true value of the technology. Understanding how it is being used, both medically and non-medically, is paramount in creating effective guidelines for practice. With the advancements in reproductive technologies, it is critical to establish global regulation of practice to ensure the safety of future generations.

## Supplementary information

ESM 1 (DOCX 30 kb)

ESM 2 (DOCX 3342 kb)

## Data Availability

Not applicable
