# Supplementary material for: Utilization of preimplantation genetic testing in the USA
Source: J Assist Reprod Genet. 2021 Apr 26;38(5):1045–53. doi: 10.1007/s10815-021-02078-4 (PMC8190209; doi:10.1007/s10815-021-02078-4)
Supplement: Supplementary file 1 — (DOCX 30 kb) [file 10815_2021_2078_MOESM1_ESM.docx]

**Supplementary figure 1**. The percentage of PGT treatment cycles by age group from 2014 to 2017.
