# Supplementary material for: Utilization of preimplantation genetic testing in the USA
Source: J Assist Reprod Genet. 2021 Apr 26;38(5):1045–53. doi: 10.1007/s10815-021-02078-4 (PMC8190209; doi:10.1007/s10815-021-02078-4)

**Supplemental Table 1A.** The states with the highest average percentage of PGT use in 2016 vs 2017.


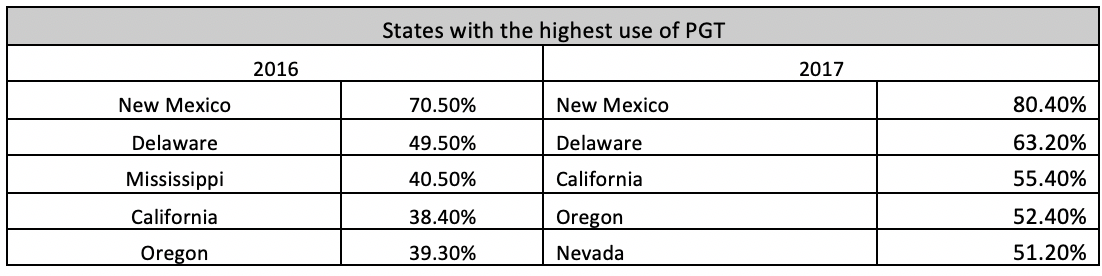


**Supplemental Table 1B.**  The states with the lowest average percentage of PGT use in 2016 vs 2017.


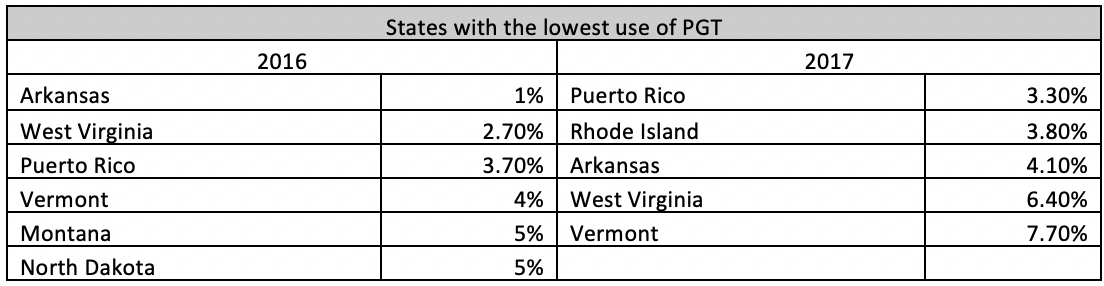


**Supplemental Table 1C.** The states that showed the greatest increase in percentage of PGT use from 2016 to 2017.


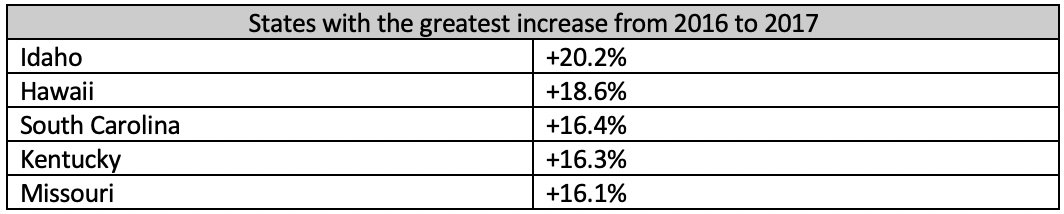

Supplement: Supplementary file 2 — (DOCX 3342 kb) [file 10815_2021_2078_MOESM2_ESM.docx]
